# Supplementary material for: A Pilot Study on the Effects of Transcranial Direct Current Stimulation on Brain Rhythms and Entropy during Self-Paced Finger Movement using the Epoc Helmet
Source: Front Hum Neurosci. 2017 Apr 25;11:201. doi: 10.3389/fnhum.2017.00201 (PMC5408787; doi:10.3389/fnhum.2017.00201)
Supplement: Supplementary file 1 [file Data_Sheet_1.zip › Complementary_results/Band_ratios_Complete_EEG/Gamma_Delta/Gamma-Delta_complete-EEG_Avg AF3-F3-F7.pdf]

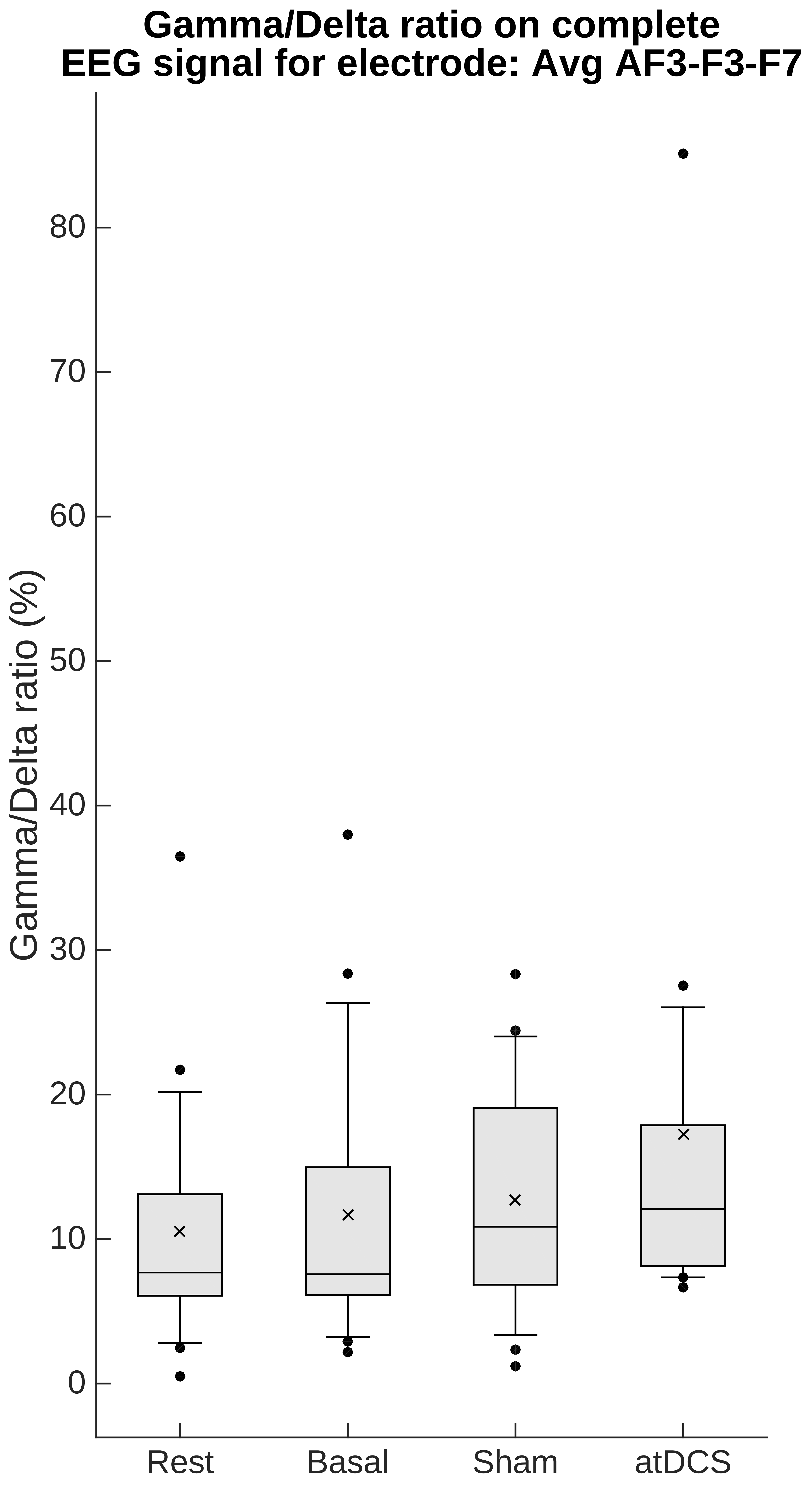
Gamma/Delta ratio on complete
EEG signal for electrode: Avg AF3-F3-F7
Gamma/Delta ratio (%)
80
70
60
50
40
30
20
10
0
Rest
Basal
Sham
atDCS
